# Physical exercise for individuals with dementia: potential benefits perceived by formal caregivers

**DOI:** 10.1186/s12877-020-01938-5

**Published:** 2021-01-06

**Authors:** A. Sampaio, I. Marques-Aleixo, A. Seabra, J. Mota, J. Carvalho

**Affiliations:** 1 CIAFEL - Research Center in Physical Activity, Health and Leisure, Faculty of Sport, University of Porto, Rua Dr. Plácido Costa 91, 4200-450 Porto, Portugal; 2 Faculty of Psychology, Education and Sport, Lusofona University of Porto, Rua Augusto Rosa, nº 24, 4000-098 Porto, Portugal

**Keywords:** Neurocognitive disorders, Caregivers distress, activities of daily life, Aging, Institutionalization, BPSD, Neuropsychiatric symptoms

## Abstract

**Background:**

The social and economic impact of dementia for the development of accessible and sustainable care for individuals with dementia (IwD). Physical exercise has been seen as a beneficial non-pharmacological therapy in the prevention and management of dementia, and possible benefits may not only impact on participants, but also indirectly on their caregivers. Thus, this quasi-experimental non-randomized study aimed to analyze the effects of an exercise intervention on functional capacity, behavioural and psychological symptoms in dementia (BPSD) and quality of life of institutionalized older adults with dementia, perceived by their formal caregivers.

**Methods:**

Sixty-four institutionalized older adults (from both genders, aged 65–93 yrs. old), clinically diagnosed with dementia, were divided into two groups: control group (CG, continued with usual care, *n* = 26) and exercise group (EG, 6-month supervised multicomponent exercise intervention, *n* = 38). Nine caregivers (female, aged 28–47 yrs. old) from nine different nursing homes, reported about their distress related to BPSD and proxy-reported about participants’ functional capacity (Katz index), quality of life (QoL-AD), BPSD (NPI) before and after 6 months of an exercise intervention (aerobic, muscular resistance, flexibility and postural exercises).

**Results:**

A two-way ANOVA, with repeated measures, revealed significant group and time interactions on Total Katz index and QoL-AD. The CG’s performance functional capacity and quality of life score worsen over time while in EG maintains these values after the exercise intervention. Moreover, formal caregiver’s distress triggered by apathy and disinhibition increased in CG while after 6 months of an exercise intervention no alterations were seen regarding these distress causes in EG. No significant main effects were observed for total NPI score or NPI distress.

**Conclusions:**

Overall results show that after the exercise intervention, IwD from the EG, was capable of preserving the functional capacity, quality of life and neuropsychiatric symptoms were attenuate, contributing to a lower load of distress for the caregivers.

**Trial registration:**

clinicaltrials.gov, NCT04095962. Retrospectively registered on 19 September 2019

## Background

Presently it is estimated that 47 million people live with dementia worldwide, and projections show that this number may increase to more than 131 million by 2050, as populations age [1]. Due to this significant prevalence, the huge social and economic impact makes dementia one of the main age-related health problems affecting society [2]. Dementia is a progressive degenerative syndrome that compromises cognitive and functional capacity essential to perform activities of daily life (ADL) autonomously [1]. Additionally, behavioral and psychological symptoms of dementia (BPSD), are commonly developed over time and tend to persist throughout the course of the disease [3], having a negative impact on the health of patients and caregivers [4]. Cognitive impairment, physical and functional dependence and mainly BPSD contribute to family caregivers’ burden and predicts the institutionalization of people with dementia (IwD) [5].

Alzheimer’s Disease International [1], reported that 33 to 50% of people with dementia, in high-income countries, live in nursing homes and more than two-thirds of care home residents have dementia. The high proportions of IwD, lack of appropriate education and training on BPSD management are physically and psychologically challenging [6] and can result in high levels of emotional exhaustion and distress of the formal caregivers [7]. Moreover, after institutionalization, formal caregivers are the individuals with whom PwD have the closest relationship, being the ones who have a better knowledge of their cognitive, physical impairments, usual BPSD, quality of life (QoL) and are aware of the progress of the disease. This sets not only the PwD but also the formal caregiver in a central position of this worldwide health problem.

Although dementia cannot be reversed, evidence suggests that poorer performance in ADL can be delayed [8]. Among others, physical exercise, i.e., planned, structured, repetitive, and purposeful physical activity, constitutes a promising intervention for IwD and has received increased attention in recent years [8]. In fact, physical exercise has been described as a non-pharmacological alternative capable to induce benefits through modulation of the brain structure and function [9]. Evidence suggests that PE promotes neurogenesis, neuroplasticity and has beneficial effects on the regulation of neurotrophic factors such as BDNF and IGF-1. Physical exercise is capable of altering important biomarkers present some forms of dementia such as in Alzheimer’s disease [9].

Indeed, exercise interventions have a positive impact on several health outcomes in IwD, including the improvement of several cardiovascular risk factors, metabolism, body composition [10] and enhancement of functional capacity [8, 11]. These factors have been described as determinant risk factors for dementia onset and its progression [12]. However, little information has been gathered on the impact of exercise on BPSD [13] incidence and the stress that those symptoms can cause to the caregivers.

Developing safe and effective exercise interventions focus on the management of BPSD, aiming at delaying the progression of declines in functional ability, and improving their QoL are urgent for IwD in nursing homes, to enhance the quality of long-term care and reduce formal caregiver burden. Despite the caregiver’s close relationship with the IwD and the unique observational role in institutional settings, their perception of exercise intervention outcomes is not highlighted, to the authors’ knowledge. Therefore, it seems relevant to determine the hypothetical positive effect of an exercise intervention in caregivers’ perception concerning functional capacity, QoL and BPSD in institutionalized older adults with mild to moderate dementia.

## Methods

### Study design

The study was designed as a multicenter quasi-experimental non-randomized study. Nine nursing homes accepted to participate in this study. The nursing homes had similar physical conditions, daily routines, number of staff and patients. The allocation of the institutions (to EG or CG) was made after an initial screening of the stage of dementia of all potential participants. The homogeneity of participants in the initial and moderate stage of dementia, for both CG and EG was the criteria used for the allocation of the institutions. Blinded assessors did the assessment and data collection. Four nursing homes implemented an exercise intervention for 6 months (Exercise Group- EG); while five did not participate in structured physical activity program and maintained their normal routine and usual care, during the same period (Control Group - CG). Usual care includes attendance at day care facilities, visits to health professionals, receipt of medication, respite care etc.

### Participants

Nine formal caregivers, in a supervising position, from nine different nursing homes, aged 28–47 years, accepted to participate in the study, proxy reporting about the effects of an exercise intervention in the IwD and reporting about the distress related with the BPSD of their care receivers. These caregivers had daily interaction with the IwD.

The eligible subject pool was restricted to older adults with the following characteristics: age ≥ 65 years, not engaged in any regular exercise training in the last year, institutionalized for more than 6 months, all diagnosed by a physician with an age-related neurocognitive disorder (dementia) at mild or moderate stage according to clinical dementia rating (CDR) [14] and lack of any diagnosed or self-reported musculoskeletal or cardiovascular disorders that contraindicate participation in moderate exercise and testing. After initial screening, formal caregivers, care receivers and institutions received a complete explanation of the purpose, risks and procedures of the study. Written informed consent was provided. The investigation was in full compliance with the Helsinki declaration [15] and the nine institutions where the intervention took place approved all methods and procedures. The Ethical Commission of the Faculty of Sports of the University of Porto also approved this study (reference: CEFADE 02.2014).

Seventy-seven older adults from both genders, aged 65–94 years, accepted to participate in this study. A total of 12 participants dropped out during the trial (15.6%). In the EG, 2 participants didn’t reach 70% of attendance rated and, in the CG, 4 dropouts were registered due to being bedridden, 3 due to death and 4 unwilling to participate in the last assessment. Sixty-four individuals from both genders, aged 65–93, concluded the study.

A 70% minimum attendance rate to the exercise sessions was required for participants in the EG. The attendance rate for the EG was calculated by dividing the number of exercise sessions completed by participants by the full amount of sessions they were expected to perform throughout the study. The attendance levels were 78.3% or over. The reasons for missing exercise sessions were acute diseases, behavioral disorders, unwillingness to participate in a particular exercise session and other reasons.

**Fig. 1:**
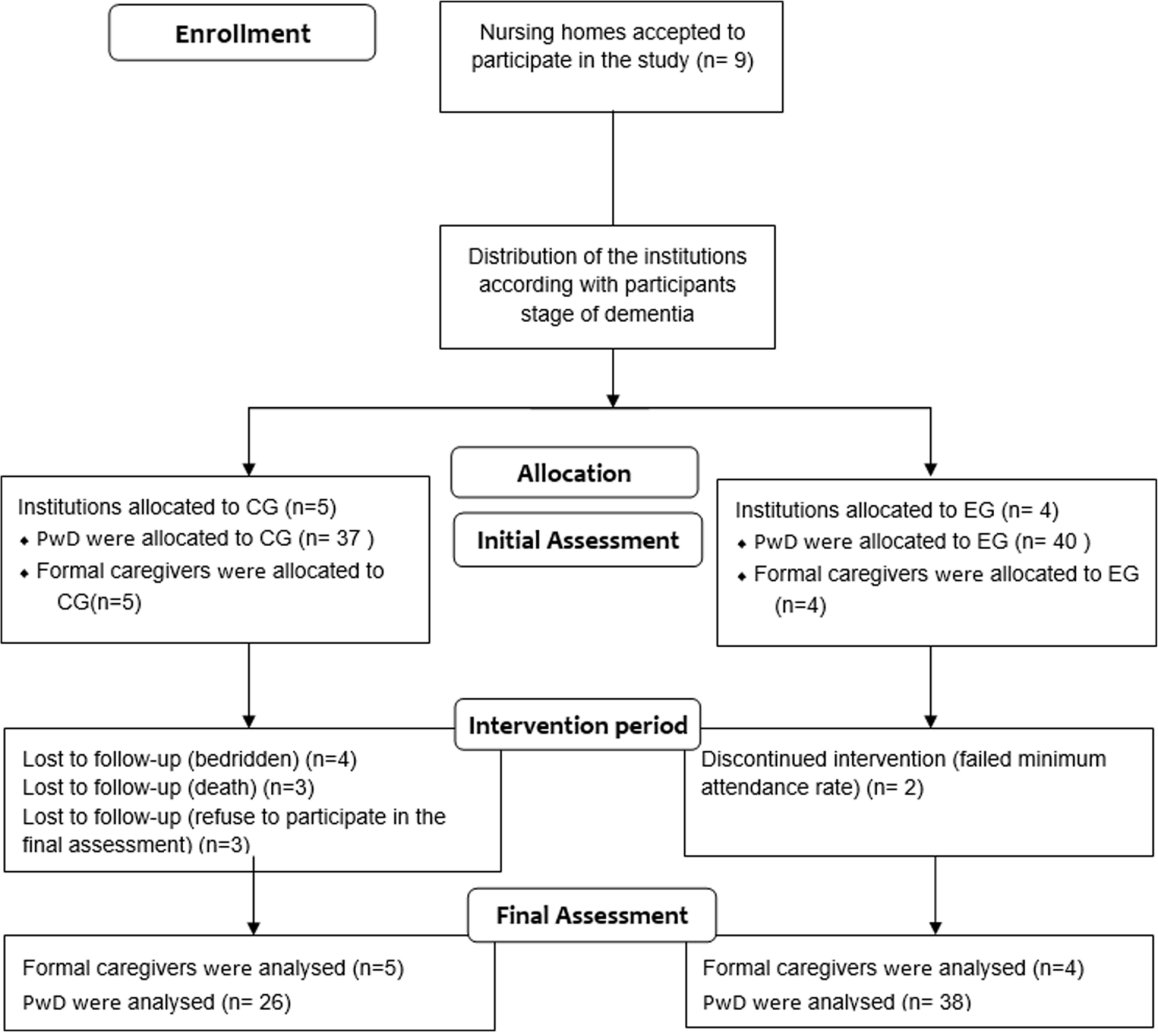
Subjects flow diagram from initial screening to the end of the study. CG, control group; EG, exercise group

The participant’s flow diagram is represented in Fig. 1.

### Exercise intervention

The EG completed a 6-month exercise program following the recommendations of the American College of Sports Medicine [16] including aerobic, muscle strengthening, flexibility, balance and postural exercises with 2 sessions per week on non-consecutive days. Sessions included 5–15 care receivers and took place in specific rooms with peaceful and pleasant background music. Sessions lasted for 45–55 min and were conducted by the same exercise trainer in all settings. The trainer is specialized in exercise for older adults. The sessions were divided into 3 main parts: warm-up (5–10 min including joint mobilization, postural and stretching exercises for general activation), specific training (30–35 min, including 15 min of moderate aerobic exercises + 15–20 min motor and muscular tasks for strength and coordination/balance training) and cool down (5 min with respiratory and stretching exercises) (see Table 1). To ensure homogeneity in the comprehension of the exercises, participation was limited to individuals in mild and moderate stages of dementia. To make the exercise program more efficient and attractive, we established regular similar routines that prioritized enjoyable and familiar exercises (such as simulating walking, running, rowing). Due to the frailty condition of the participants and for safety reasons, sessions were mainly chair-based and routines of functional exercises with low coordination requirements were emphasized so care receivers could achieve the session’s goals.

**Table 1:** Description of the exercise intervention

Sessions Main-parts	Warm-up	Aerobic Resistance	Strength /Balance & Coordination	Stretching & Cool down
**Exercises**	General activation + joint mobilization + Stretching exercises E.g. Stationary walk, range of motion exercises for the shoulders, hip, knees, and ankles; chest stretch; sit and reach; knee to chest; seated forward bend	Aerobic exercises in stationary position or chair based. E.g. Stationary walk & chair-based exercises: - Seated jumping jacks; - Seated running; - Seated rowing; - Seated tap dance.	Strength exercises involving mainly major upper and lower body muscle groups + balance & coordination exercises. E.g. one leg-stand; heel-to-toe walk, bodyweight shifting.	Stretching exercises involving mainly major upper and lower body muscle groups + respiratory exercises. E.g. chest stretch; sit and reach; knee to chest; seated forward bend; deep breathing, Huff cough, Diaphragmatic breathing.
**1st & 2nd months**				
**Time**	10 min	5 + 5 + 5 min	15 min	5 min
**Training Load**		55–65% HRmax	Strength exercises: 4 × 10–15 reps Static balance + coordination: 2 exercises	
**3rd & 4th months**				
**Time**	8 min	10 + 5 min	22 min	5 min
**Training** **Load**		60–75% HRmax	Strength exercises: 5–6 × 10–15 reps Static & Dynamic balance + coordination: 2 exercises	
**5th & 6th months**				
**Time**	5 min	15 min	30 min	5 min
**Training** **Load**		65–80% HRmax	Strength exercises: 6 × 10–15 reps Static & Dynamic balance + coordination:2–3 exercises	

### Sociodemographic and clinical characteristics

Formal caregivers’ supervisors and care receivers’ characteristics are given in Table 2.

**Table 2:** Characteristics of the participants at baseline

**CAREGIVERS**	**Exercise Group** (*n* = 4)	**Control Group** (*n* = 5)	**p**^**a**^
**Age (years)**	37.75 ± 8.14	36.40 ± 7.54	0.806
**Geriatric working experience (years)**	9.50 ± 3.51	8.60 ± 2.51	0.683
**CARE RECEIVERS**	**Exercise Group** (n = 38)	**Control Group** (n = 26)	**p**^**a**^
**Age (years)**	77.29 ± 8.60	80.15 ± 2.80	0.179
**Men, No. (%)**	14 (36.8%)	5 (19.2%)	0.169
**CDR (points)**	1.18 ± 0.90	1.13 ± 0.63	0.808
**MMSE (points)**	18.11 ± 4.81	17.77 ± 5.07	0.268
**Educational level (years)**	2.60 ± 1.72	2.27 ± 1.54	0.381
**Neurocognitive Disorder due to, No. (%)**			0.259
Alzheimer’s Disease	16 (42.1%)	18 (69.2%)	
Vascular Disease	6 (15.8%)	3 (11.5%)	
Parkinson’s Disease	1 (2.6%)	0 (0%)	
Lewy Bodies Disease	1 (2.6%)	0 (0%)	
Unspecified	14 (36.8%)	5 (19.2%)	
**Diagnosis, No. (%) (Others than NCD)**			
Hypertension	14 (36.8%)	4 (15.4%)	0.045*
Heart Disease	9 (23.7%)	2 (7.7%)	0.141
Diabetes mellitus	5 (13.2%)	5 (19.2%)	0.463
Osteoporosis	3 (7.9%)	3 (11.5%)	0.650
**Blood Pressure (mmHg)**			
Systolic	126.72 ± 22.50	128.81 ± 22.10	0.718
Diastolic	76.47 ± 14.64	76.73 ± 17.31	0.950
**Physical Fitness**			
Lower Strength (reps)	8.95 ± 4.31	9.96 ± 4.16	0.352
Upper-Strength (reps)	9.71 ± 5.18	11.77 ± 4.11	0.096
Lower Flexibility (cm)	20.86 ± 10.43	19.69 ± 12.82	0.691
Agility/ Dynamic balance (sec)	19.33 ± 10.86	20.36 ± 12.52	0.726
Upper Flexibility (cm)	43.53 ± 16.44	42.88 ± 10.34	0.861
Aerobic Endurance (steps)	58.61 ± 32.82	69.46 ± 34.72	0.209

^a^ Student’s t-test or Mann Whitney for continuous variables; Chi-squared or Fisher’s exact

### Outcome measures

All following measures were assessed at baseline and 6 months of exercise intervention. CDR test, Senior Fitness Test (SFT) and Mini-Mental State Examination (MMSE) were performed by the care receivers. The care receivers Katz index, quality of life - Alzheimer disease scale (QoL-AD) and Neuropsychiatric Inventory (NPI) were proxy-reported by the caregivers.

### Dementia stage

The CDR test [14] was used only at baseline to allocate the care receivers according to their cognitive stage. CDR is an instrument that assesses the existence and prevalence of the various stages of dementia. It comprises 6 cognitive-behavioral items covering memory, orientation, judgment and problem solving, community activities, home and hobbies, and personal care. The cut-off points were CDR = 1 (mild dementia stage) and CDR = 2 (moderate dementia stage).

### Physical fitness

The SFT [17] battery is considered a reliable instrument for assessing physical fitness in older adults (≥ 60 years old) including older people with cognitive impairment [18]. The test items included: chair-stand test - to assess lower-body strength; arm curl test - to measure upper-body strength; 2-min step test - to assess aerobic endurance; chair sit-and-reach test - to assess lower-body flexibility; back scratch test - to assess upper-body flexibility; and 8-ft up-and-go test - to assess agility and dynamic balance.

### Cognitive function

The MMSE [19] was used for a global cognitive evaluation. This instrument is clinically used to assess cognitive mental status, detect and follow the course of mental illness and can also be used as a research tool to screen for cognitive disorders and follow cognitive changes in epidemiological studies. It assesses orientation, attention, immediate and short-term recall, language and the ability to follow simple verbal and written instructions. Furthermore, it provides a total score that categorizes the individual on a scale of cognitive function ranging from 0 to 30 [19]. MMSE normative values consider the subject’s educational level. Operational cut-off values for the Portuguese population are 22 (for 0 to 2 years of literacy), 24 (for 3 to 6 years of literacy) and 27 (for more than 6 years of literacy) [20].

### Functional capacity

Katz index [21], one of the most used instruments for measuring the ability to perform ADL, was used to proxy-report the physical functioning of the IwD. Katz index includes 6 items: bathing, dressing, transferring, feeding, incontinence, toileting and the sum of all items to calculate the Katz total. Independence levels for the ADL questions are recorded on a scale of 0 to 4, where 0 represents dependence and 5 represents complete independence [22].

### Quality of life

The QoL-AD [23] was used to measure the IwD QoL. The questionnaire included 13 items: physical health, energy, mood, living situation, memory, family, marriage, friends, self as a whole, ability to do chores, ability to do things for fun, financial situation, and QoL as a whole. The QoL-AD provides the participant and caregiver reports of the participant’s QoL and is scored on a 4-point Likert scale ranging from 1 to 4 (excellent), with total scores ranging between 13 and 52 points.

### Behavioral and psychological symptoms of dementia

The Neuropsychiatric Inventory (NPI) [24] is an instrument that proxy-reports IwD changes in BPSD over time. The NPI originally assessed 10 behavioral domains (delusions, hallucinations, agitation, dysphoria, anxiety, apathy, irritability, euphoria, disinhibition, and aberrant motor behavior). Two more domains have been added since its development: night-time behavioral disturbances and appetite and eating abnormalities [24]. For each of the 12 behavioral symptoms on the NPI, caregivers rated the level of distress they experienced, due to IwD behavioral symptoms, on a scale from 1 (low) to 5 (extreme). The NPI Distress score (NPI-D) [25] is the sum of these 12 ratings (range 0–60). In the present study, we used the Portuguese version published in the Book of Scales of the Study Group on Brain Ageing and Dementia [26].

### Statistical analysis

Results were expressed as either means (standard deviations) or proportions (Table 2). Differences between groups at baseline were tested using unpaired sample t-tests, mann-whitney, and chi-square tests. The intervention effects results were expressed as a percentage of the baseline values of the CG and were examined by repeated-measures analysis of variance ANOVA (2 times [initial and final] × 2 groups [EG and CG]). When ANOVA revealed significant (time, group or interaction), Bonferroni post hoc tests were performed to evaluate pairwise differences. Between-group differences for all outcomes were adjusted to age, as a confounder. Partial eta squared values (η2p) were reported to quantify the effect sizes.

Significance level was set at 0.05 throughout the analyses. Statistical analyses were performed using SPSS 26.0.

### Sample size

The required sample size was calculated with G*Power (Version 3.1.9.2, Heinrich Heine University of Duesseldorf) [27]. A power analysis based on a formulation of 80% power, a moderate effect size of 0.25 for NPI, and a significance level of 0.05 for a two-tailed test deemed that a sample of 34 subjects (17 per group) was sufficient to address the research questions.

## Results

### Characteristics of the participants

Sociodemographic and clinical characteristics of the participants are summarized in Table 2. The sample included 9 formal caregivers’ supervisors, all female and with geriatric nursing assistant certification. The IwD that participated in the study were 70.3% females and all presented a neurocognitive disorder (NCD). Hypertension, minor heart condition, diabetes mellitus and osteoporosis were the other main diagnoses besides the NCD. The CDR showed that the average of the care receivers was in the moderate stage of dementia for both groups. No significant differences were observed for the total cognitive function score assessed by MMSE between groups. There were also no significant differences at baseline between groups were observed in physical fitness variables.

The baseline results showed significant differences in incontinence (functional capacity component) between EG and CG, reported by caregivers.

Caregivers did not perceive other significant differences between groups at baseline, including BPSD-score, BPSD-distress, functional capacity components and QoL (Table 3).

**Table 3:** BPSD-score, BPSD-caregivers´ distress, functional capacity and QoL results at baseline

	Exercise Group (*n* = 4 about 38 IwD)	Control Group (n = 5 about 26 IwD)	p^**a**^
**BPSD – Score (points)**			
Delusions	0.97 ± 1.92	1.15 ± 2.75	0.759
Hallucinations	0.61 ± 1.48	1.31 ± 3.37	0.259
Agitation	1.79 ± 3.01	2.35 ± 3.07	0.473
Dysphoria	1.11 ± 2.04	1.54 ± 2.67	0.465
Anxiety	1.89 ± 2.72	2.58 ± 3.08	0.354
Euphoria	0.84 ± 2.39	1.81 ± 3.64	0.205
Apathy	1.39 ± 2.33	1.27 ± 2.55	0.839
Disinhibition	1.37 ± 2.73	2.42 ± 4.09	0.220
Irritability	1.82 ± 3.27	2.31 ± 3.67	0.576
Aberrant motor activity	1.45 ± 2.71	1.04 ± 2.46	0.540
Night behavioral disturbances	1.13 ± 2.22	1.38 ± 2.77	0.687
Appetite/eating abnormalities	1.55 ± 3.00	2.35 ± 3.81	0.355
NPI Total	15.92 ± 18.42	21.5 ± 26.66	0.325
**BPSD – Caregivers´ Distress (points)**			
Delusions	0.82 ± 1.31	0.77 ± 1.37	0.890
Hallucinations	0.53 ± 1.03	0.73 ± 1.37	0.499
Agitation	1.11 ± 1.71	1.38 ± 1.58	0.510
Dysphoria	0.82 ± 1.41	1.12 ± 1.58	0.430
Anxiety	1.11 ± 1.39	1.5 ± 1.48	0.281
Euphoria	0.47 ± 1.06	0.92 ± 1.44	0.155
Apathy	0.79 ± 1.21	0.96 ± 1.46	0.609
Disinhibition	0.79 ± 1.28	1.04 ± 1.58	0.490
Irritability	0.97 ± 1.46	1.19 ± 1.47	0.560
Aberrant motor activity	0.87 ± 1.19	0.65 ± 1.26	0.492
Night behavioral disturbances	0.68 ± 1.30	0.88 ± 1.48	0.568
Appetite/eating abnormalities	0.89 ± 1.52	1.23 ± 1.75	0.417
**NPI Total - Distress**	8.87 ± 10.01	11.19 ± 14.13	0.444
**Functional Capacity (points)**			
Bathing	1.74 ± 0.89	1.81 ± 0.75	0.740
Dressing	2.00 ± 1.09	1.88 ± 1.07	0.677
Toileting	2.47 ± 0.80	2.00 ± 1.20	0.062
Transferring	2.53 ± 0.80	2.08 ± 1.13	0.066
Incontinence	2.61 ± 0.82	2.12 ± 0.91	0.029*
Feeding	2.71 ± 0.87	2.19 ± 1.39	0.070
**Total Katz**	14.11 ± 3.90	12.00 ± 4.95	0.062
**Quality of Life (points)**			
**Total QoL-AD (points)**	24.13 ± 5.07	22.27 ± 5.30	0.162

^a^ Student’s t-test; * *p* ≤ 0.05

### Physical fitness

**Fig. 2:**
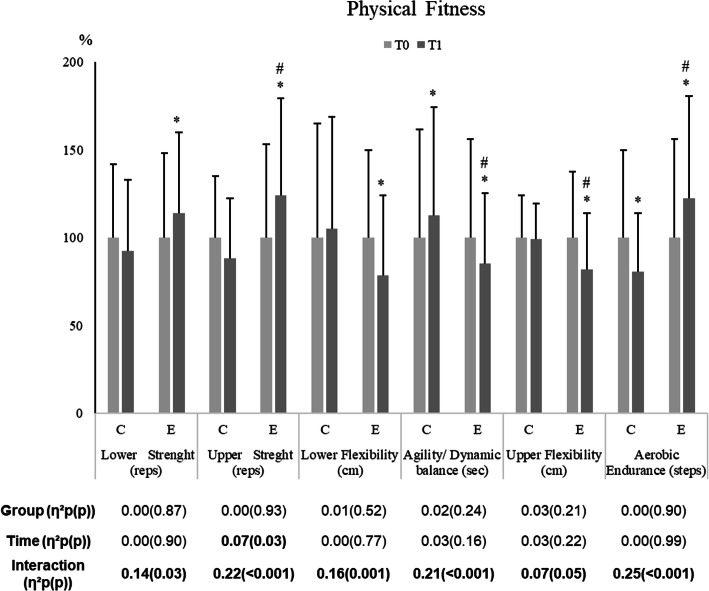
Effects of a multicomponent training intervention on physical fitness components of institutionalized older adults with dementia; n = 26 in the CG and n = 38 in the EG. Results were expressed as a percentage of T0. *vs T0; # vs EG (*p* ≤ 0.05 The effect size (partial eta squared) and significant (*p* ≤ 0.05) main effects of Group, Time and/or their interaction are indicated. T0 baseline values, before the exercise intervention; T1 after 6 months of exercise intervention. Results adjusted to age

As seen in Fig. 2, for all the physical fitness components, a significant group by time interaction was found. Six months of an exercise intervention improved the EG, physical fitness components whereas no alteration was seen in CG (except for agility-balance and aerobic endurance in which a decrease in performance was observed).

### Behavioral and psychological symptoms of dementia score

**Fig. 3:**
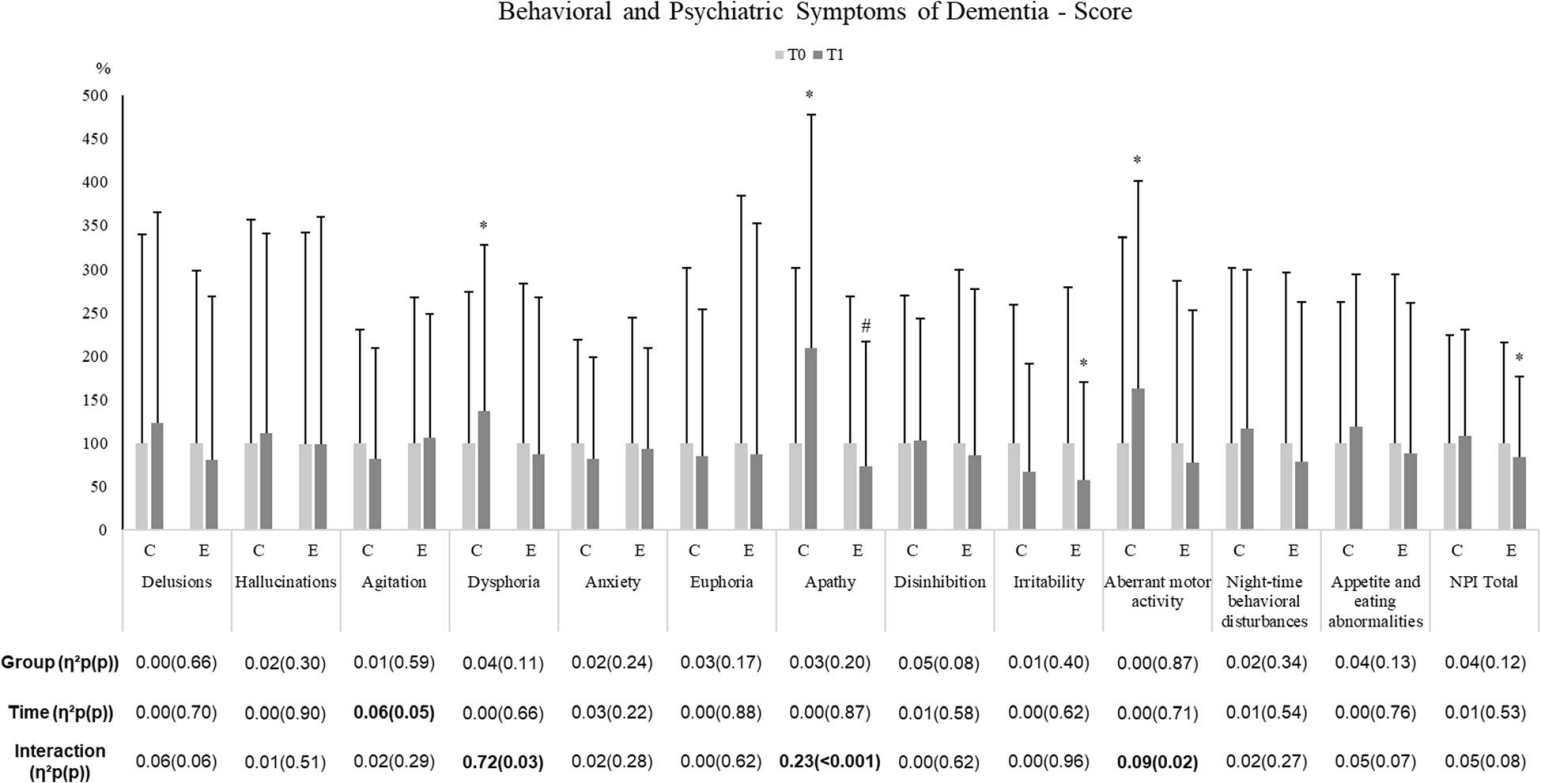
Effects of an exercise intervention on proxy-rated outcomes of neuropsychiatric symptoms score (NPI) of institutionalized older adults with dementia. *n* = 26 in the CG and *n* = 38 in the EG. Results were expressed as a percentage of T0. *vs T0; ^#^ vs CG (*p* ≤ 0.05). The effect size (partial eta squared) and significant (*p* ≤ 0.05) main effects of Group, Time and/or their interaction are indicated. T0 baseline values, before the exercise intervention; T1 after 6 months of the exercise intervention. Results adjusted to age

Alterations in BPSD scores following the exercise intervention (EC) and control period (CG) are shown in Fig. 3 and Table 4. The analyses for dysphoria, apathy and aberrant motor activity indicated a significant group by time interaction effect. In CG, caregivers’ proxy-reported a higher presence of dysphoria, apathy, and aberrant motor activity comparing baseline and T1 after 6 months, whereas these neuropsychiatric symptoms score were preserved in EG (except for a significant decreased in apathy score).

**Table 4:** Physical Fitness, BPSD-score, BPSD-caregivers´ distress, functional capacity and QoL after 6 months

VARIABLE	Exercise Group (*n* = 4 about 38 IwD)	Control Group (*n* = 5 about 26 IwD)	Mean Differences (CI 95%); ***P*** value
**Physical Fitness**			
Lower Strength (reps)	10.47 ± 3.75	9.19 ± 4.07	0.2 (−1.8 to 2.2); 0.87
Upper-Strength (reps)	12.32 ± 4.98	10.38 ± 4.0	0.1 (− 2.2 to 2.4); 0.93
Lower Flexibility (cm)	16.39 ± 9.50	20.69 ± 12.56	−1.8 (−7.4 to 3.8); 0.52
Agility/ Dynamic balance (sec)	16.49 ± 7.78	22.98 ± 12.50	−1.8 (−7.4 to 3.8); 0.24
Upper Flexibility (cm)	35.70 ± 13.86	42.50 ± 8.71	−3.1 (−8.5 to 2.2); 0.21
Aerobic Endurance (steps)	71.71 ± 34.11	56.08 ± 23.01	0.9 (−14.5 to 16.6); 0.90
**BPSD – Score (points)**			
Delusions	0.79 ± 1.82	1.42 ± 2.77	−0.3 (−1.4 to 0.9); 0.66
Hallucinations	0.61 ± 1.6	1.46 ± 3.00	−0.6 (−1.8 to 0.6); 0.30
Agitation	1.89 ± 2.55	1.92 ± 2.99	−0.4 (−1.7 to 1.0); 0.59
Dysphoria	0.97 ± 1.99	2.12 ± 2.93	−1.0 (−2.1 to 0.2); 0.11
Anxiety	1.76 ± 2.20	2.12 ± 2.30	−0.8 (−2.1 to 0.5); 0.24
Euphoria	0.74 ± 2.23	1.54 ± 3.06	−1.0 (−2.4 to 0.4); 0.17
Apathy	1.03 ± 1.98	2.65 ± 3.41	−0.8 (−2.1 to 0.4); 0.20
Disinhibition	1.18 ± 2.61	2.50 ± 3.38	−1.3 (−2.8 to 0.2); 0.08
Irritability	1.05 ± 2.04	1.54 ± 2.89	−0.5 (−2.0 to 0.8); 0.40
Aberrant motor activity	1.13 ± 2.54	1.69 ± 2.50	−1.0 (− 1.4 to 1.3); 0.87
Night behavioral disturbances	0.89 ± 2.06	1.62 ± 2.52	−0.5 (−1.6 to 0.6); 0.34
Appetite/eating abnormalities	1.37 ± 2.67	2.81 ± 4.10	−1.3 (− 3.0 to 0.4); 0.13
**NPI Total**	13.42 ± 14.61	23.38 ± 26.10	−8.4 (−19.1 to 2.30); 0.12
**BPSD – Caregivers´ Distress (points)**			
Delusions	0.68 ± 1.07	1.04 ± 1.54	−0.1 (−0.7 to 0.5); 0.72
Hallucinations	0.63 ± 1.10	0.96 ± 1.60	−0.2 (− 0.9 to 0.4); 0.44
Agitation	1.34 ± 1.34	1.42 ± 1.65	−0.2 (−1.0 to 0.5); 0.53
Dysphoria	0.76 ± 1.40	1.23 ± 1.30	−0.5 (−1.2 to 0.2); 0.13
Anxiety	1.26 ± 1.45	1.35 ± 1.52	−0.3 (−1.1 to 0.4); 0.33
Euphoria	0.37 ± 0.89	0.85 ± 1.38	−0.5 (−1.1 to 0.4); 0.07
Apathy	0.63 ± 1.20	1.50 ± 1.45	−0.5 (−1.2 to 0.1); 0.07
Disinhibition	0.71 ± 1.25	1.54 ± 1.50	−0.6 (−1.3 to 0.1); 0.07
Irritability	0.87 ± 1.44	0.92 ± 1.44	−0.2 (− 0.9 to 0.5); 0.57
Aberrant motor activity	0.76 ± 1.29	0.96 ± 1.18	−0.04 (− 0.5 to 0.6); 0.90
Night behavioral disturbances	0.63 ± 1.29	1.19 ± 1.65	−0.4 (−1.1 to 0.2); 0.19
Appetite/eating abnormalities	0.84 ± 1.14	1.42 ± 1.8	−0.6 (−1.4 to 0.2); 0.16
**NPI Total - Distress**	8.63 ± 8.60	13.46 ± 12.71	−0.6 (−1.3 to 0.1); 0.14
**Functional Capacity (points)**			
Bathing	1.76 ± 0.88	1.88 ± 0.86	−0.9 (− 0.5 to 0.3); 0.69
Dressing	2.11 ± 1.06	1.77 ± 1.03	0.2 (−0.3 to 0.8); 0.37
Toileting	2.47 ± 0.80	1.85 ± 1.00	0.6 (0.1 to 1.0); 0.02*
Transferring	2.68 ± 0.84	1.73 ± 0.96	0.7 (0.2 to 1.2); 0.003*
Incontinence	2.61 ± 0.82	1.92 ± 0.98	0.6 (0.1 to 1.0); 0.01*
Feeding	2.79 ± 0.81	2.04 ± 1.25	0.6 (0.1 to 1.2); 0.02*
**Total Katz (points)**	14.42 ± 3.80	11.19 ± 4.57	2.7 (0.5 to 4.9); 0.02*
**Quality of Life**			
**Total QoL-AD (points)**	26.38 ± 4.01	22.47 ± 5.04	2.3 (0.4 to 4.3); 0.02*

*Significant (*p* ≤ 0.05); CI: confidence interval; Results adjusted to age

### Behavioral and psychological symptoms of dementia **-** distress

**Fig. 4:**
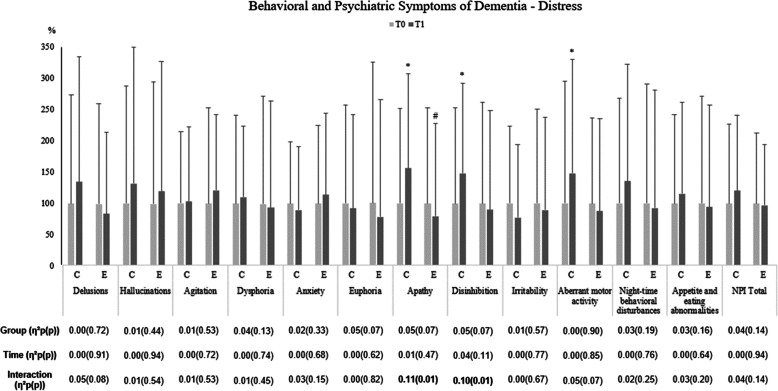
Effects of an exercise intervention on caregivers’ neuropsychiatric symptoms distress of institutionalized older adults with dementia. n = 26 in the CG and n = 38 in the EG. Results were expressed as a percentage of T0. *vs T0; ^#^ vs CG (*p* ≤ 0.05). The effect size (partial eta squared) and significant (*p* ≤ 0.05) main effects of Group, Time and/or their interaction are indicated. T0 baseline values, before the exercise intervention; T1 after 6 months of the exercise intervention. Results adjusted to age

BPSD caregivers´ distress scores at baseline and following exercise intervention are shown in Fig. 4 and Table 4. For the apathy and disinhibition dimensions, a significant group by time interaction was observed. Among CG, the caregivers´ distress due to apathy and disinhibition increased whereas among EG decreased at follow-up compared with baseline. A significant interaction for the disinhibition and aberrant motor activity dimensions showed a decreased in CG not seen in EG comparing baseline and T1.

### Functional capacity and quality of life

**Fig. 5:**
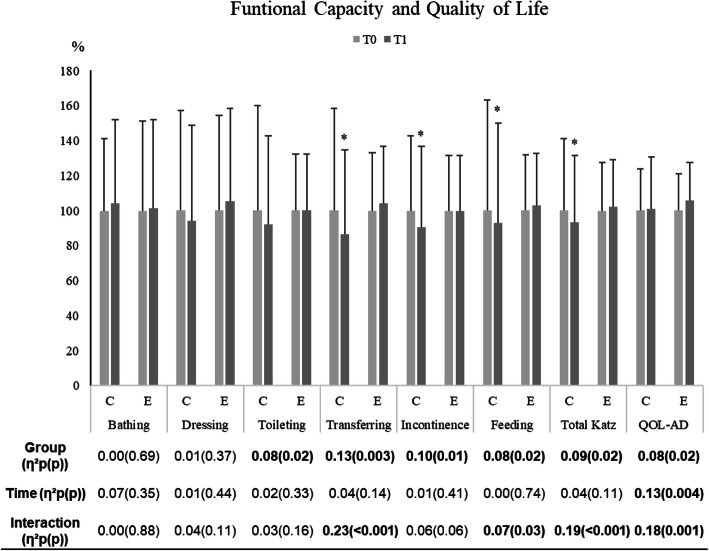
Effects of an exercise intervention on proxy-rated functional capacity of and QoL outcomes of institutionalized IwD. Data are mean ± standard deviation; n= 26 in the control group (CG) and n= 38 in the exercise group (EG). Results were expressed as a percentage of T0. *vs T0; ^#^ vs EG (*p* ≤ 0.05). The effect size (partial eta squared) and significant (*p* ≤ 0.05) main effects of Group, Time and/or their interaction are indicated. T0 baseline values, before the exercise intervention; T1 after 6 months of the exercise intervention. Results adjusted to age

Figure 5 shows the proxy-rated outcomes of the functional capacity and QoL at baseline and following an exercise intervention for each group. For the transferring, feeding total Katz and QoL-AD, a significant interaction between groups and time was observed. Among CG, the values of these variables decreased comparing the baseline and the follow-up while in EG remained unaltered after the exercise intervention.

## Discussion

Exercise programs may be relevant for IwD living in institutional settings since they spend an extended period in those settings [28] with a high rate of functional decline [29] and they are frequently physically inactive [28, 30]. Although exercise interventions are low cost, feasible and can be easily implemented, there is a lack of physical activity opportunities in institutions [31].

The caregivers as the people with more contact with IwD are the most appropriate ones to evaluate the outcomes of an exercise intervention. Therefore, the present study highlighted proxy-rated outcomes and the caregiver’s perception of exercise intervention as a possible strategy to mitigate symptoms and alleviate disease progression in institutionalized IwD.

The hypothesis that a 6-month exercise program can promote positive proxy-rated outcomes concerning its effects on BPSD and functional capacity in institutionalized older adults with mild to moderate dementia was confirmed. This study also tested the hypothesis if a 6-month exercise program can promote a positive the importance of an exercise intervention on QoL, this hypothesis was also confirmed.

The present study attempted to adapt an exercise program for IwD, living in institutional settings to the physical exercise recommendations of American College of Sports Medicine (ACSM) and American Heart Association (AHA) for older adults [16]. The exercise intervention integrated several important physical abilities to perform the ADLs and involving playful and social group activities. Corroborating other studies that applied for similar training programs in terms of duration, frequency and type of population [11, 30] improvements were seen in all the physical fitness components evaluated in the group that participate in the 6 months’ exercise intervention (Fig. 2). It is possible that these positive results were linked with low scores seen before the intervention. These results also suggested that the exercise intervention was adjusted to the characteristics of our population and enough to induce physical adaptations. This underlines that the adaptation of the physical activity recommendations to this population, allows them to improve their levels of physical fitness.

Although some studies revealed positive outcomes of an exercise intervention on cognitive function, most of these studies were conducted with older adults without dementia [32, 33]. In fact, in this special population physical exercise showed controversial results, with studies suggesting no alterations in general cognition in comparison with the control group [8]. Our intervention did not significantly alter general cognition in comparison with the control group. Probably, as suggested by Lautenschlager & Cox [34] to improve brain function and cognition, exercise intervention should be more extended in time.

BPSD are an intrinsic feature of dementia, often treated with antipsychotics. Current person-centered philosophies of care in dementia encourage non-pharmacological therapies as an alternative and/or complementary interventions for minimizing BPSD [35], including exercise programs. The literature points out controversial results, while Forbes et al. [8] found no clear evidence of the positive effects of an exercise intervention on BPSD, other authors affirm that exercise interventions can be beneficial to reduce some of the BPSD [36, 37]. Besides the controversy, these studies agree that further work is needed to comprehend the potential of exercise as non-pharmacological therapy to manage BPSD [36]. Evidence suggests that exercise may affect various BPSD in different ways [37]. Indeed, the effects of exercise seem to be more beneficial on depressed mood, agitation and reduce “wandering” [37]. The results from our study seem to be in line with this evidence, generally EG maintains their BPSD scores similar to the scores reported on the baseline, while the CG worsen depression, apathy, and aberrant motor behavior scores (Fig. 3). Importantly, in our study, decreased of the total BPSD in the EG was observed after the 6 months’ exercise intervention.

To our knowledge, no studies have addressed the caregiver’s perspective about the impact of exercise intervention in their BPSD distress. In our study, formal caregiver’s distress triggered by apathy and lack of inhibition increased in CG while after 6 months of an exercise intervention no alterations were seen regarding these distress causes in EG (Fig. 4). Evidence suggests that disruptive behaviors and low ADL levels among residents with dementia expose formal caregivers to demanding physical and emotional distress [7], leading to poorer QoL [38, 39]. Thus, our results could suggest that exercise programs in institutionalized IwD may be useful as a strategy for BPSD- caregivers´ distress management. Strategies to alleviate the burden felt by formal caregivers leads to higher job satisfaction, increase their QoL and consequently improved staff attitudes and caring behaviors and, over time, residents’ well-being [40].

Functional status is related to institutionalization [41]. Among other reasons, in most cases, older adults move to a nursing home when their functional capacity is diminished, affecting their independency to perform ADL [42]. In institutional settings, assistance in ADL for older adults are often delivered in a standardized and depersonalized way that undermines independence [43]. Particularly in IwD, fewer opportunities to perform ADL and the lack of physical activity opportunities exacerbate the functional decline in institutional settings [44]. Corroborating this evidence, the present study showed a progressive decline in the total functional capacity score and some of their domains including transferring, feeding, and incontinence outcomes in the group without exercise intervention. Conversely the EG was capable of preserving their total functional capacity after the 6 months of exercise intervention (Fig. 5). Other studies have verified that exercise programs implemented in institutions can induce positive outcomes concerning the functional capacity of IwD [8, 45, 46].

It has been suggested that in institutional settings the participation in a wide range of activities improves the QoL of IwD [47]. In fact, activity engagement may contribute to the pleasure and enjoyment, the sense of connection and belonging and retain a sense of autonomy and personal identity [48]. Importantly, caregivers also consider aspects such as social relationships, physical movement, attachment and affect, control over life, and contributing to the community as important for IwD QoL [49]. A 3 months aerobic exercise randomized control trial [50] and 16 weeks multi center exercise program [4], both for older adults with Alzheimer’s Disease shown some evidence that exercise programs can improve QoL. Corroborating with these results, our study has shown positive results on QoL following 6 months of EG (Fig. 5).

BPSD are commonly associated with a reduction in the QoL for the older adults with dementia [51] and increase of caregiver stress [47, 51]. Although the majority of the studies regarding functional capacity and QoL evaluation of institutionalized IwD have been partially or fully reported by the formal caregivers (proxy-rated reported), the fact that this is the perspective of the caregiver has not been highlighted. In fact, it seems relevant to emphasize their perception since dementia care can contribute (due to disruptive behavior and the limited capacity of performing ADL) to the burden of formal caregivers [47]. Additionally, to the authors’ knowledge, no previous studies have explored the effects of 6 months’ exercise intervention on BPSD score and caregivers’ distress in institutional settings. Higher levels of stress and poorer levels of well-being of formal caregiver’s impact negatively on the quality of care they provide and consequently have a negative effect on institutionalized IwD’s well-being [52]. Therefore, interventions for residents with dementia perceived as positive by the formal caregiver may increase the well-being both of themselves and, by extension, those they care for. The results of our study showed that formal caregivers perceived some of the benefits of the engagement to exercise program in institutionalized IwD.

The small sample size and the lack of randomization for group assignment are relevant limitations of the study. Due to the low number of participants in this study restricted the statistical analysis to be adjusted to only one potential confounder: age. However, the recruitment of individuals clinically diagnosed with dementia willing to commit to a 6-month intervention demanded the participation of several institutions, with similar characteristics, leading to a very challenging recruitment process. Although the data collection was done by blinded assessors, due to the nature of the intervention, it wasn’t possible to blind nursing homes or proxy-reporters regarding the allocation group.

Furthermore, psychotropic medication has been associated with several adverse outcomes and given the widespread use this type of medication by IwD, future studies should assess the impact of the use of psychotropic medication, in exercise interventions.

## Conclusions

In sum, this study confirms that a 6-month exercise program can promote positive proxy-rated outcomes on functional capacity and BPSD in institutionalized older adults with mild to moderate dementia. Moreover, this study suggests that an exercise intervention in institutionalized IwD may be useful as a strategy of BPSD- caregivers’ distress management, alleviating the burden felt by formal caregivers. After the intervention, improved QoL in IwD has also been observed. These results indicate that the engagement in the exercise program in institutionalized IwD as positive. This can be relevant for better well-being of both IwD and formal caregivers in institutional settings.

## Data Availability

The data that support the findings of this study are available from the corresponding author upon reasonable request.

## Abbreviations

ACSM: American College of Sports Medicine
ADL: Activities of Daily Life
ANOVA: Repeated-measures analysis of variance
BPSD: Behavioral and Psychological Symptoms of Dementia
BPSD-distress: Behavioral and Psychological Symptoms of Dementia Distress
BPSD-score: Behavioral and Psychological Symptoms of Dementia Score
CDR: Clinical Dementia Rating
CG: Control Group
EG: Exercise Group
IwD: Individuals with Dementia
MMSE: Mini-Mental State Examination
NCD: Neurocognitive Disorder
NPI: Neuropsychiatric Inventory
NPI-D: Neuropsychiatric Inventory Distress score
SFT: Senior Fitness Test
QoL-AD: Quality of Life - Alzheimer Disease Scale
QOL: Quality of Life
